# Deletion of *Kncn* Does Not Affect Kinocilium and Stereocilia Bundle Morphogenesis and Mechanotransduction in Cochlear Hair Cells

**DOI:** 10.3389/fnmol.2018.00326

**Published:** 2018-09-11

**Authors:** Qun Hu, Li Guo, Jie Li, Chenmeng Song, Lisheng Yu, David Z. Z. He, Wei Xiong

**Affiliations:** ^1^Tsinghua-IDG/McGovern Institute for Brain Research, School of Life Sciences, Tsinghua University, Beijing, China; ^2^Department of Otolaryngology, Peking University People’s Hospital, Beijing, China; ^3^Department of Biomedical Sciences, School of Medicine, Creighton University, Omaha, NE, United States

**Keywords:** *kncn*, hair cell, kinocilium, hair bundle, mechanotransduction, cochlea

## Abstract

Auditory hair cells possess stunning cilia structure that composes of a bundle of stereocilia for mechano-electrical transduction and a single kinocilium for guiding the polarity of hair bundle towards maturation. However, the molecules underlying kinocilium function have not yet been fully understood. Hence, the proteins involved in hair bundle development and function are of a large interest. From a fine microarray analysis, we found that *kinocilin* (*Kncn*) was enriched in hair cell specific expression profile. Consistently, it has been reported that KNCN was a protein mainly located in the kinocilium of hair cells in the inner ear. However, the hypothesis that KNCN is a kinocilium protein has not been validated in mice with *Kncn* gene perturbed. In this study, we generated *Kncn* knockout mouse lines by CRISPR/Cas9 technique and further examined the morphology and function of cochlear hair cells. Our results showed that there was no obvious hearing loss in the knockout mice, determined by audiometry. Histological study demonstrated that the inner ear and hair cell structure were intact. Especially, there was no deficit of mechanotransduction (MET) in cochlear outer hair cells (OHCs). In summary, our work suggests that KNCN is not essential for kinocilium-oriented hair bundle function in cochlear hair cells.

## Introduction

Cochlear hair cells are neuroepithelial cells characterized by hair bundles on their apical surface (Flock, 1971; Fettiplace and Hackney, 2006; Hudspeth, 2013). Hair cells transduce mechanical stimuli into electrical activity (Hudspeth and Corey, 1977). The site of hair cell transduction is at the hair bundle, an array of modified microvilli or stereocilia arranged in a staircase-like shape with the longest stereocilia juxtaposed next to the kinocilium (Pickles et al., 1984; Kachar et al., 2000). Adjacent stereocilia are connected along their shafts by side links, whereas tip links, formed by cadherin 23 and protocadherin 15 (Siemens et al., 2004; Kazmierczak et al., 2007), extend from the apex of each stereocilium to the side of its taller neighbor (Pickles et al., 1984; Kachar et al., 2000; Goodyear et al., 2005). The stereocilia contain bundles of uniformly polarized actin filaments with the barbed ends pointing toward the stereociliary tips (Tilney and Tilney, 1986). The filaments contain β- and γ-actin and are cross-linked by espin, plastin1 and T-plastin (Tilney et al., 1989; Zine et al., 1995; Zheng et al., 2000; Daudet and Lebart, 2002). To guide hair bundle in position, the kinocilium is the key structure to polarize the apex and guide bundle formation in sensory hair cells.

Despite the fact that the kinocilium has long been considered as a transient embryonic feature without any functional significance in the adult cochlear hair cells, it is critical for the development of hair bundle polarity and orientation. At the onset of hair bundle morphogenesis, a single microtubular kinocilium is localized in the center of the apical cell surface covered with microvilli. The kinocilium subsequently moves to the destination side of cuticular plate and the first row of stereocilia next to the kinocilium start to elongate, as well as the following adjacent rows of stereocilia (Frolenkov et al., 2004). The kinocilium is important for hair bundle development and maturation but not contributing in mechanotransduction (MET; Hudspeth and Jacobs, 1979). As a primary cilium, it presents with a microtubular composition of 9 + 0, 9 + 2 or 8 + 1 (Kikuchi et al., 1988; Kelley et al., 1992; Sobkowicz et al., 1995), which implies a dynamic assembly of microtubule there. Although the kinocilium remains in adult mammalian vestibular hair cells and in all hair cells of nonmammals, the kinocilium degenerates in auditory hair cell upon maturation of the hair bundle. For example, the kinocilium starts to retract from postnatal day 8 (P8) and be completely gone at P12, in the mouse cochlear hair cells. Mutations lead to the loss of the kinocilium in hair cells followed by the degeneration of the sensory cells have been described in zebrafish (Tsujikawa and Malicki, 2004). Loss of kinociliary links is considered a cause of abnormal polarity of hair bundles in PCDH15-CD2 deficient mice (Webb et al., 2011). However, the factors including genes involved in kinocilium development and dynamics are not known completely.

The identification of genes commonly or preferentially expressed in the two types of cochlear hair cells, the inner hair cells (IHCs) and the outer hair cells (OHCs), is a powerful approach for deciphering the molecular organization and subsequent characterization of gene function of the two types of hair cells. Using microarray technique, we detected high expression of kinocilin (*Kncn*) in adult IHCs and OHCs (Liu et al., 2014). *Kncn*, encoding Kinocilin, was first detected in the kinocilia of vestibular and auditory hair cells at embryonic days 14.5 and 18.5, respectively (Leibovici et al., 2005). KNCN was still present in the kinocilium in the mature vestibular hair cells (Leibovici et al., 2005). In mature auditory hair cells, KNCN was present at the cuticular plate, at the base of each stereocilium. KNCN was also expressed by the pillar cells and Deiters cells, that both contain prominent transcellular and apical bundles of microtubules (Leibovici et al., 2005). Immunogold staining and biochemical study showed that the KNCN localized in microtubule-rich regions, where vesicles and cargos were actively transported (Leibovici et al., 2005). However, the function of KNCN has not been characterized up-to-date.

In this study, we examined the effect of *Kncn* deletion on stereocilia morphogenesis and hearing loss in mice. Hair bundle morphology was observed by fluorescence microscopy and scanning electron microscopy (SEM). MET of the stereocilia was evaluated by recording mechanically activated membrane current from hair cells of *Kncn* knockout mice. Our goal is to determine whether KNCN plays an important role in stereocilia/kinocilium-based bundle morphogenesis and maintenance, MET, and hair cell survival. Our results show that hair bundle has no obvious defect in hair cells of *Kncn* knockout mice. In parallel, the MET and hair cell survival are normal in the knockout mice.

## Materials and Methods

### Animal Care

The use of animals was approved and the experimental procedures were regulated by the Institutional Animal Care and Use Committee of Tsinghua University.

### Generation of *Kncn* Knockout Mice

The *Kncn* knockout mice were generated in our animal center by CRISPR/Cas9 technology. The *Kncn* sgRNA was designed using the online CRISPR toolbox^1^ (Zhang Feng Lab) which targeted the Exon 1 of *Kncn*. T7-Cas9 plasmid (kindly provided by Dr. Yichang Jia at Tsinghua University) was linearized by Xba I (NEB), and purified by HiPure Gel Pure DNA mini Kit (D2111-02, Magen, China), and worked as the template (1 μg) for *in vitro* transcription by mMESSAGE mMACHINE T7 ultra transcription kit (Ambion AM1345, Thermo Fisher Scientific, Waltham, MA, USA). The Cas9 mRNA was purified by MEGAclear Kit (Ambion AM1908, Thermo Fisher Scientific, Waltham, MA, USA) and dissolved in the microinjection buffer (MR-095-10F, Millipore, Burlington, MA, USA). Both T7 promoter and targeting sgRNA sequences were added into sgRNA backbone template by PCR amplification using designed primers (Kncn-sgRNA F and R). Then the PCR product was purified with gel using HiPure Gel Pure DNA mini Kit (D2111-02, Magen, China) and used as the template (100 ng) for *in vitro* transcription using T7 RNA Polymerase (10881767001, Roche, Germany). The sgRNA were purified by using phenol: chloroform extraction and alcohol precipitation method, and then dissolved in the microinjection buffer (MR-095-10F, Millipore, Burlington, MA, USA). The C57BL/6 mouse line was used for generating knockout. Cas9 mRNA (20 ng/μl) and sgRNA (10 ng/μl) targeting *Kncn* gene were mixed and injected into the cytoplasm of the fertilized eggs. The sequences of forward and reverse primers used for targeting sgRNA are listed in Supplementary Table S1.

### Genotyping

To genotype the littermate mice, the genomic DNA was extracted from the tails and the fragment around the gRNA target site was amplified by PCR and the genotypes were further determined by sequencing. The sequences of forward and reverse primers used for PCR genotyping of wild-type and *Kncn* knockout mice are listed in Supplementary Table S1.

### RT-PCR and Q-PCR Analysis

Total RNA of different tissue was extracted using HiPure Universal RNA Kit (Magen, R4130-02) according to the manufacturer’s protocol. Reverse transcription was carried out using HiScript II Q RT SuperMix for qPCR (+gDNA wiper) Kit (Vazyme, R223-01) according to the manufacturer’s protocol. Polymerase chain reaction using 1 μl cDNA template for 10 μl system was performed using Phanta Max Super-Fidelity DNA Polymerase (Vazyme, P505-d1). Then the agarose gel electrophoresis was performed to semi-quantify the mRNA level of *Kncn*. Q-PCR was carried out using ChamQ SYBR Q-PCR Master Mix (Low ROX Premixed) Kit (Vazyme, Q331-02) according to the manufacturer’s protocol. The sequences of RT-PCR and Q-PCR primers were listed in Supplementary Table S1.

### *In situ* Hybridization

The inner ears of P4 mice were used for cryosection with 12 μm thickness. Generation of the RNA probe and *in situ* hybridization (ISH) were performed as described previously (Grillet et al., 2009). The RNA probe complementary to part of mouse *Kncn* cDNA (NCBI: NM_001039124) was amplified using primers containing T7 and T3 promoter (Kncn-ISH-F/Kncn-ISH-R) for *in vitro* transcription using T7 RNA Polymerase (10881767001, Roche, Germany) and T3 RNA Polymerase (11031163001, Roche, Germany).

### Scanning Electron Microscopy

Inner ears were dissected out in phosphate buffer (0.1 M Na_2_HPO_4_·12H_2_O, 0.1 M NaH_2_PO_4_·2H_2_O, PH 7.4) and transferred into fixative buffer (2.5% glutaraldehyde, 0.1 M phosphate buffer). A hole was poked at the apex to let the fixative flush through the cochlear labyrinth before the sample was fixed overnight at 4°C. The inner ears were washed with phosphate buffer for 10 min three times and fine-dissected to remove the spiral ligament, Reissner’s membrane and tectorial membrane. Samples were dehydrated by 30-min incubation in 10/20/30/50/70/80/90/100/100% ethanol, followed by 100/100/100% tertiary butanol before freeze drying (Hitachi ES-2030) and gold coating (Hitachi E-1010). The samples were imaged with FEI Quanta 200.

### Immunostaining

The inner ear was dissociated from P4 mice. Cochleae of *Kncn* knockout and wild-type mice were collected in PBS and placed into PBS with 4% PFA for 30 min at room temperature (RT). The cochlea tissues were washed with PBS and blocked by using PBSTx (PBS + 0.5% Triton X-100) with 4% bovine serum albumin (BSA) for 1 h at RT. The samples were incubated with primary antibodies (in PBSTx + 1% BSA) overnight at 4°C. After 3× washes with PBS, samples were further incubated with secondary antibody and phalloidin in PBSTx overnight at 4°C. Lastly, samples were again washed for six times with PBS. The samples were imaged with a deconvolution microscope (Delta Vision Elite, GE, USA). The following antibodies were used: KNCN antibody (1:200, rabbit, NBP2-48844, Novus Biological), Acetyl-alpha Tubulin (Lys40) Monoclonal Antibody (6-11B-1; 1:800, mouse, #32-2700, Thermo Fisher Scientific, Waltham, MA, USA), Goat anti-rabbit IgG (H + L) Highly Cross-Adsorbed Secondary Antibody Alexa Fluor 488 (1:2,000, A-11029, Invitrogen, Thermo Fisher Scientific, Waltham, MA, USA), Goat anti-mouse Alexa Fluor 568 Phalloidin (1:2,000, A12380, Invitrogen, Thermo Fisher Scientific, Waltham, MA, USA).

### Electrophysiology

Hair cells were recorded using whole-cell patch-clamp technique as described previously (Xiong et al., 2012). Briefly, the basilar membrane (BM) with hair cells was acutely dissociated from P7 mice. The dissection solution contained (in mM): 141.7 NaCl, 5.36 KCl, 0.1 CaCl_2_, 1 MgCl_2_, 0.5 MgSO_4_, 3.4 L-Glutamine, 10 glucose and 10 H-HEPES (pH 7.4). Then the BM was transferred into recording chamber with recording solution containing (in mM): 144 NaCl, 0.7 NaH_2_PO_4_, 5.8 KCl, 1.3 CaCl_2_, 0.9 MgCl_2_, 5.6 glucose and 10 H-HEPES (pH 7.4). The BM was used for electrophysiological recording within 1 h. An upright microscope (Olympus BX51WI) was used to observe hair cells. Patch pipettes were made from borosilicate glass capillary (Sutter, BF150-117-10) with a pipette puller (Sutter, P-2000) and polished with a microforge (Narishige, MF-830) to resistances of 3–5 MOhm. Intracellular solution contains (in mM): 140 KCl, 1 MgCl_2_, 0.1 EGTA, 2 Mg-ATP, 0.3 Na-GTP and 10 H-HEPES, pH 7.2). The OHCs in the apical, middle, and basal part of the cochleae were recorded. Whole cell currents were sampled at 100 KHz with a patch-clamp amplifier (HEKA EPC 10 USB + Patchmaster software). Hair cells were voltage-clamped at −70 mV. The hair bundle was deflected by a fluid-jet pipette with a tip diameter of 5–10 μm that was positioned ~5 μm to the hair bundle to evoke maximum MET currents. The stimulation was in 40 Hz sinusoidal wave delivered from a 27-mm-diameter piezoelectric disc driven by a homemade piezo amplifier.

### Auditory-Brainstem Response (ABR) Measurements

The adult mice were tested to evaluate hearing threshold by click auditory brainstem response (click ABR) as described previously (Chen et al., 2016). Before measurement, the mouse was anesthetized by i.p. injection of pentobarbitone. Then the mouse was transferred into a sound-proof chamber (Shengnuo, Shanghai) for audiometry. The audiometric evaluation was done with an auditory workstation (Tucker-Davis Technologies RZ6 system). The electrodes were placed into the mouse sub-dermally. The ground electrode was inserted in the back near the hind leg, and the reference electrode was just behind the pinna, and the active electrode was inserted at the vertex. A close-field speaker (TDT MF1) was placed onto the external ear canal through a conducting tube. A balanced click or pure tone stimuli (MF1 Speaker response range: 1–50 kHz) were applied per second, each with a duration of 0.1 ms, starting at 90 dB SPL and decreasing at 10 dB SPL step in intensity. Stimuli and recordings were performed with the BioSigRZ software provided with the TDT workstation. The number of acquisition trials was set to 512 for averaging.

### Statistical Analysis

All the data are presented as mean ± SD or SEM depending on the cell number. Excel software (Microsoft) and Igor pro software (Wavemetrics) were used to analyze the data. Statistical significance was determined using a two-tailed, unpaired Student’s *t*-test, by which the result was noted in each panel.

## Results

### *Kncn* Gene Expression in Auditory Epithelium

**Figure 1:**
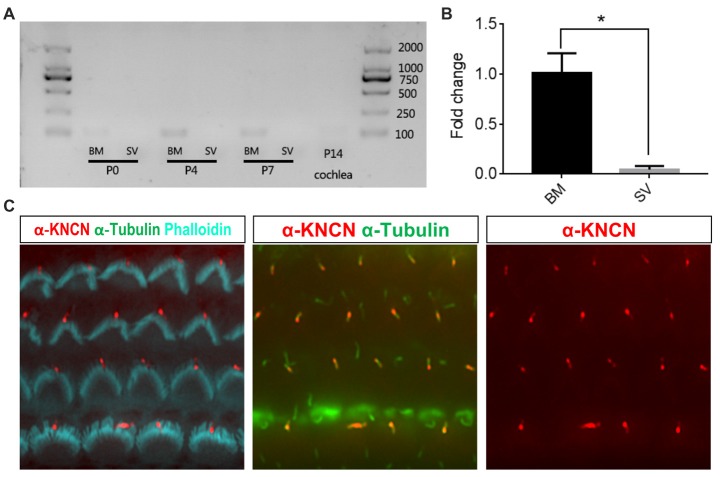
Kinocilin (*Kncn*) expression in the auditory epithelium of mouse inner ear. **(A)** RT-PCR of *Kncn* mRNA from the basilar membrane (BM) and the stria vascularis (SV) at P0, P4 and P7, as well as the cochleae at P14. The positive band for *Kncn* was 139bp that was amplified by qPCR primers. The RT-PCR experiments were repeated three times. **(B)** Statistics of quantitative mRNA level of *Kncn* from P7 BM and SV by qPCR. Data were presented as mean ± SD, *n* = 3. *P < 0.05. **(C)** Immunostaining of KNCN protein on cochlear hair cells from P4 wild-type mouse.

To check the temporal expression pattern of *Kncn* gene in the hearing organ, we first performed RT-PCR to evaluate expression level of mRNAs collected from cochlear tissues at P0, P4, P7 and P14 ages. The results showed that *Kncn* was expressed in BM in adequate amount but with high specificity at first week, especially at P4. There were not visible expression in other tissues, even in stria vascularis (SV; Figure 1A and Supplementary Figure S1A). We further examined the expression level of *Kncn* by qPCR, which showed a significant *Kncn* expression largely in BM but much lesser in SV (Figure 1B). The KNCN antibody also demonstrated a strong signaling on kinocilia of hair cells from P4 mice (Figure 1C). These data were consistent with our microarray data (Liu et al., 2014) and Leibovici et al. (2005) that have showed a concentrated expression of KNCN protein in mouse inner ears. Consistently, we also observed similar staining pattern of KNCN in young adult cochleae at 1 month age, in which expressed in the pericuticular necklace of IHCs and in rootlets of OHCs (data not shown). However, we did not see *Kncn* expression in kidney, liver and heart (Supplementary Figure S1A), as reported by Leibovici et al. (2005). We further performed ISH to determine cell type specific distribution of *Kncn* mRNA in the inner ear at P4, from which the expression of *Kncn* was detected in the hair cells (Supplementary Figure S1B).

### Generation of *Kncn* Knockout Mice

**Figure 2:**
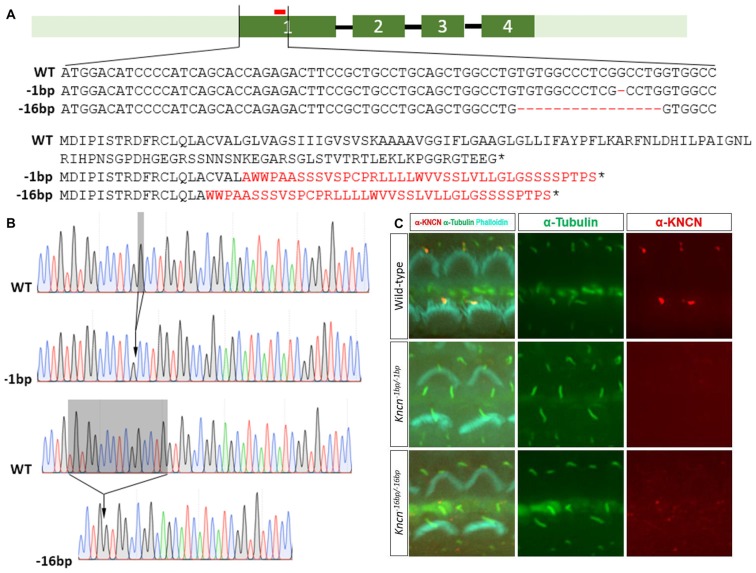
Generation and characterization of *Kncn* knockout mice. **(A)** Schematic drawing of generation of *Kncn* knockout mice via CRIPSR/Cas9 methods. Targeting sites of *Kncn* gene was in the middle of Exon 1. Two of knockout lines were used in this study, in which one line possessed a 1bp deletion (*Kncn*^−1bp/−1bp^) and another line had a 16bp deletion (*Kncn*^−16bp/−16bp^), both resulting in frame shift mutation of encoded protein with early stop. Red bar marked location of the targeting guide RNA (gRNA). Dash indicted deleted nucleotide and asterisk noted a premature stop codon. The mis-translation of amino acids was shown in red. **(B)** Sequencing chromatograms of wild-type, *Kncn*^−1bp/−1bp^ and *Kncn*^−16bp/−16bp^ homozygous mice. **(C)** Characterization of KNCN defect in *Kncn*^−1bp/−1bp^ and *Kncn*^−16bp/−16bp^ homozygous mice by KNCN antibody.

Together with previous study, our *in vitro* and *ex vivo* observation in the cochleae and hair cells suggested that *Kncn* gene might play a role in hair bundle development and/or function. To explore the physiological role of KNCN in hearing and transduction, *Kncn* knockout mouse lines were generated using the CRISPR/Cas9 genome editing technique. The mouse *Kncn* gene contains four exons, with the start and stop codon localizing in the first and last exon, respectively (Figure 2A). A guide RNA (gRNA) targeting site in the middle of exon 1 was designed to make gene disruption. Genomic DNA sequencing result showed that five insert/deletions (Indels) have been successfully introduced into exon 1 of *Kncn* of founder mice, in which we chose two genotypes for further breeding (Figure 2A). The two mutant mouse lines hosted deletions of 1 bp (*Kncn*^−1bp/−1bp^) and 16 bp (*Kncn*^−16bp/−16bp^), respectively after the start codon validated by Sanger sequencing (Figures 2A,B). These changes caused premature translational stops, including a truncated protein of 20 amino acids and a truncated protein of 16 amino acids (Figure 2A). The CRISPR-generated F0 *Kncn* heterozygous mice were further crossed with wild type C57BL/6 mice to bring up the F2 mice with homozygous genotypes. The KNCN antibody could not recognize the kinocilia structure anymore (Figure 2C), which demonstrated that the two *Kncn* knockout mouse lines were successfully generated using CRISPR/Cas9 genome editing technique. In the following work, we used *Kncn*^−16bp/−16bp^ mice for detailed analysis for reason of data consistency.

### Hair Bundle Structure Is Normal in *Kncn*^−16bp/−16bp^ Mice

**Figure 3:**
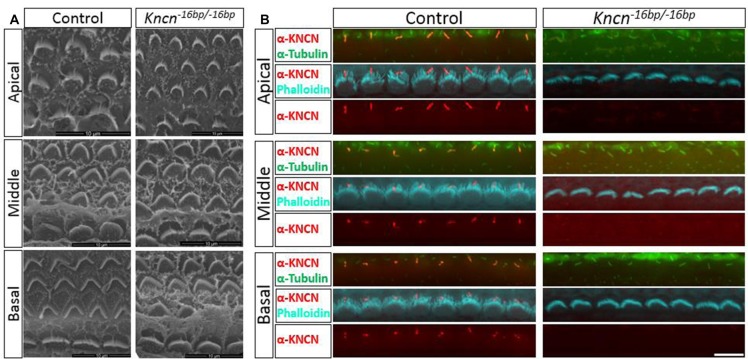
Microstructure of hair bundle of the *kncn*^−16bp/−16bp^ mice at P4. **(A)** Scanning electron microscopy (SEM) of hair bundles in hair cells from the apical, middle, basal turn. Kinocilia was developed normally at this age. **(B)** Imaging of immunostaining micrograph of stereocilia and kinocilia of hair cells in the apical, middle and basal turn. Scale bar: 10 μm **(A)**, 5 μm **(B)**.

Given the previous report that *Kncn* is expressed in the cochlea, specifically in hair cell kinocilia (Leibovici et al., 2005), we used SEM to examine the ultrastructure of kinocilia at higher resolution. At P4, an age that animals still keep kinocilia structure, the hair bundle structure of *Kncn*^−16bp/−16bp^ mice represented by SEM was rather indistinguishable comparing to that of the control mice, no matter where in apical, middle and basal turns (Figure 3A). Moreover, there was no obvious defect of stereocilia and kinocilia revealed by SEM in *Kncn*^−16bp/−16bp^ mice. Similarly, the stereocilia and kinocilia of *Kncn*^−16bp/−16bp^ mice recognized by phalloidin and tubulin antibody were normally organized in different cochlear turns (Figure 3B). These data demonstrated that the hair bundle structure of *Kncn*^−16bp/−16bp^ mice was still normal in morphology.

### MET of OHCs Is Intact in *Kncn*^−16bp/−16bp^ Mice

**Figure 4:**
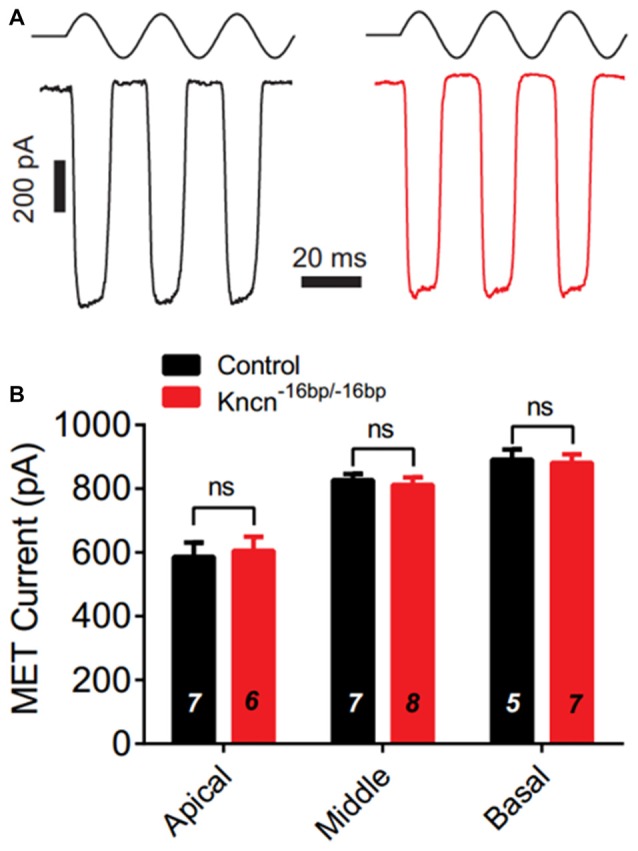
Mechanotransduction (MET) current of outer hair cells (OHCs) is not affected in *Kncn*^−16bp/−16bp^mice. **(A)** Representative traces showed MET currents induced by fluid jet stimulation in the cochlear OHCs of control (black) and *Kncn*^−16bp/−16bp^ (red) mice. In this case, middle cochlear OHCs were recorded. **(B)** Summary of saturated MET currents from all recorded OHCs from control and *Kncn*^−16bp/−16bp^ mice similar to that in **(A)**. Apical: control, 586.4 ± 44.88 pA and *Kncn*^−16bp/−16bp^, 605.8 ± 43.32 pA. Middle: control, 827.2 ± 18.7 pA and *Kncn*^−16bp/−16bp^, 811.7 ± 23.67 pA. Basal: control, 891.2 ± 32 pA and *Kncn*^−16bp/−16bp^, 881.6 ± 26.21 pA. *N* number was marked on each bar. There was no significant difference. Data were presented as mean ± SEM.

We speculated that lack of KNCN might not induce obvious structure deficit but affect hair bundle related function. To further investigate whether KNCN contributes to the MET, we used whole-cell patch-clamp technique to record OHCs in *Kncn*^−16bp/−16bp^ mice. To elicit MET response, the hair bundle was deflected by a fluid-jet that is considered to generate saturated mechanical stimulation. The MET currents of *Kncn*^−16bp/−16bp^ OHCs were not affected comparing to the control at apical, middle and basal coils (Figures 4A,B). Together, our electrophysiological study suggested that the OHCs of *Kncn*^−16bp/−16bp^ mice had normal MET responsibility.

### *Kncn*^−16bp/−16bp^ Mice Show No Obvious Hearing Loss

**Figure 5:**
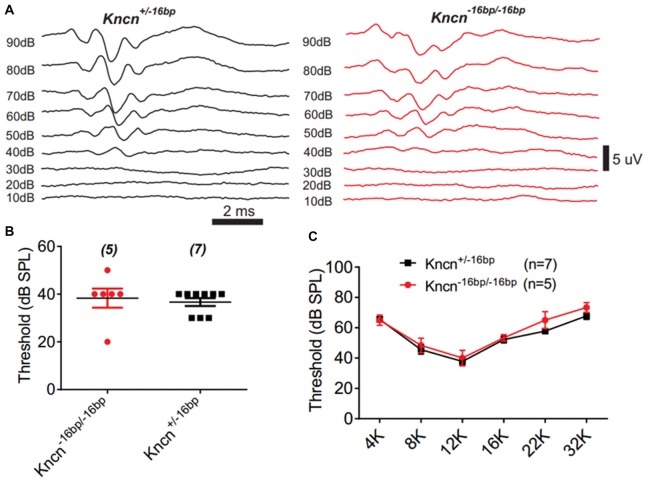
Auditory-brainstem response (ABR) measurement of *Kncn*^−16bp/−16bp^ mice. **(A)** Representative ABR traces of littermate control and *Kncn*^−16bp/−16bp^ mice at 3 month in response to a click sound stimulation. The hearing threshold of *Kncn*^−16bp/−16bp^ mice was not altered obviously comparing to the wild type control. **(B)** Statistics of click ABR threshold of wild-type and *Kncn*^−16bp/−16bp^ mice from recordings similar to **(A)**. Control (36.7 ± 1.7 dB SPL, *n* = 7); *Kncn*^−16bp/−16bp^ (38.3 ± 4 dB SPL, *n* = 5). Data were presented as mean ± SEM. **(C)** Statistics of pure-tone ABR threshold of control and *Kncn*^−16bp/−16bp^ mice. Data were presented as mean ± SEM. And no significance observed.

The auditory function of *Kncn* knockout mice was further evaluated by audiometry. The click ABR measurement at the age of 3 months showed no obvious hearing loss in *Kncn*^−16bp/−16bp^ mice. The hearing threshold of *Kncn*^−16bp/−16bp^ mice was similar to their heterozygous siblings, both around 40 dB (Figures 5A,B). Similarly, the hearing was still comparable to control when knockout mice get old at 7 months (data not shown). We further examined the pure-tone ABR and found no frequency specific defect in *Kncn*^−16bp/−16bp^ mice (Figure 5C). It prompted that omitting KNCN likely did not affect the auditory transduction.

## Discussion

KNCN expression was detected in both cochlear and vestibular hair cells (Liu et al., 2014; Scheffer et al., 2015). Its ortholog was also detected in zebrafish hair cells (Barta et al., 2018). KNCN is thought to play a role in stabilizing dense microtubular networks or in vesicular trafficking and is thus speculated that deletion and/or mutations of *Kncn* would lead to abnormal kinocilium morphogenesis as microtubules are major components in kinocilium, stereocilium and apical surface of hair cells and supporting cells (Leibovici et al., 2005). We generated *Kncn* knockout mice to examine its role in hair bundle formation and maintenance as well as in MET. Two different types of knockout mice with different insert/deletions in the *Kncn* gene were generated. We examined bundle morphology and MET in cochlear hair cells from neonatal mice. Based on our detailed study on one knockout line, *Kncn*^−16bp/−16bp^, deletion of *Kncn* has no effect on formation of kinocilium and morphogenesis of stereociliary bundles (Figure 3) and MET (Figure 4), and KNCN deletion does not lead to phenotypical changes in hearing threshold in terms of ABR at 3-month-old mice (Figure 5). We also examined ABR-based hearing threshold in mice at 7 months and observed no abnormality of hearing either, suggesting that KNCN may not be involved in vesicular trafficking in hair cells and spiral ganglion neurons.

Although *Kncn* is highly expressed in all hair cells and KNCN is detected initially in kinocilium during stereocilia bundle morphogenesis and late in cuticular plate of hair cells and some supporting cells (Leibovici et al., 2005), it appears that KNCN is not essential for kinocilium formation and morphogenesis of hair bundles. Lack of auditory defects also suggests that it is not a key player for stabilizing dense microtubular networks or in vesicular trafficking in hair cells and supporting cells. We entertain two possibilities for the lack of phenotypical changes in kinocilia and stereocilia bundle morphology. First, although KNCN is a component of kinocilium, it does not play a major role in kinocilium structure and function. This is likely as deletion of KNCN results in no change in kinocilium- and stereocilium-based bundle formation. Although kinocilium in cochlear hair cells is only transiently existed in cochlear hair cells and regresses from developing auditory hair cells before hair cells are functionally and morphologically mature before P12, the presence of kinocilium in nascent hair cells (examined at P4) suggests that kinocilium formation is not altered after *Kncn* is deleted. The conclusion is the same for vestibular hair cells although kinocilium is present in vestibular hair cells in both nascent and adult mice. The second possibility is that KNCN is an important component but the lack of phenotype is due to compensation of KNCN by other microtubules-related proteins that are not appreciated yet. As such a highly dynamic structure, many components responsible for its microtubule assembly and disassembly, polarization, kinociliary link regulation and ciliary transportation are still elusive (Sobkowicz et al., 1995; Zine et al., 1995; Zheng et al., 2000; Daudet and Lebart, 2002; Frolenkov et al., 2004; Tsujikawa and Malicki, 2004; Goodyear et al., 2005). KNCN may be only one of them as a complicated kinociliary machinery that can recruit alternative components for kinocilium function when KNCN is omitted. Hence, it is important that many aspects concerning kinocilium constitution and function need a further and thorough study.

## Author Contributions

QH, LG, JL and CS carried out the experiments. LY, DH and WX planned the work and wrote the manuscript.

## Conflict of Interest Statement

The authors declare that the research was conducted in the absence of any commercial or financial relationships that could be construed as a potential conflict of interest.

## Footnotes

^1^http://crispr.mit.edu/

## Supplementary Material

FIGURE S1 Analysis of *Kncn* expression. **(A)** RT-PCR study of *Kncn* mRNA level in different tissues from P7 mice. The positive band for *Kncn* was 405bp. Abbreviation: M, maker; BM, basilar membrane; SV, stria vascularis; Cere, cerebellum; H, heart; K, kidney; L, liver. **(B)** ISH analysis of *Kncn* mRNA expression in cochlear sections from P4 mice. Panels showed the sense and anti-sense probe staining in the organ of Corti and hair cells.

TABLE S1 List of primers used in the study.
