# Supplementary figures and images for: Deletion of Kncn Does Not Affect Kinocilium and Stereocilia Bundle Morphogenesis and Mechanotransduction in Cochlear Hair Cells
Source: Front Mol Neurosci. 2018 Sep 11;11:326. doi: 10.3389/fnmol.2018.00326 (PMC6141681; doi:10.3389/fnmol.2018.00326)

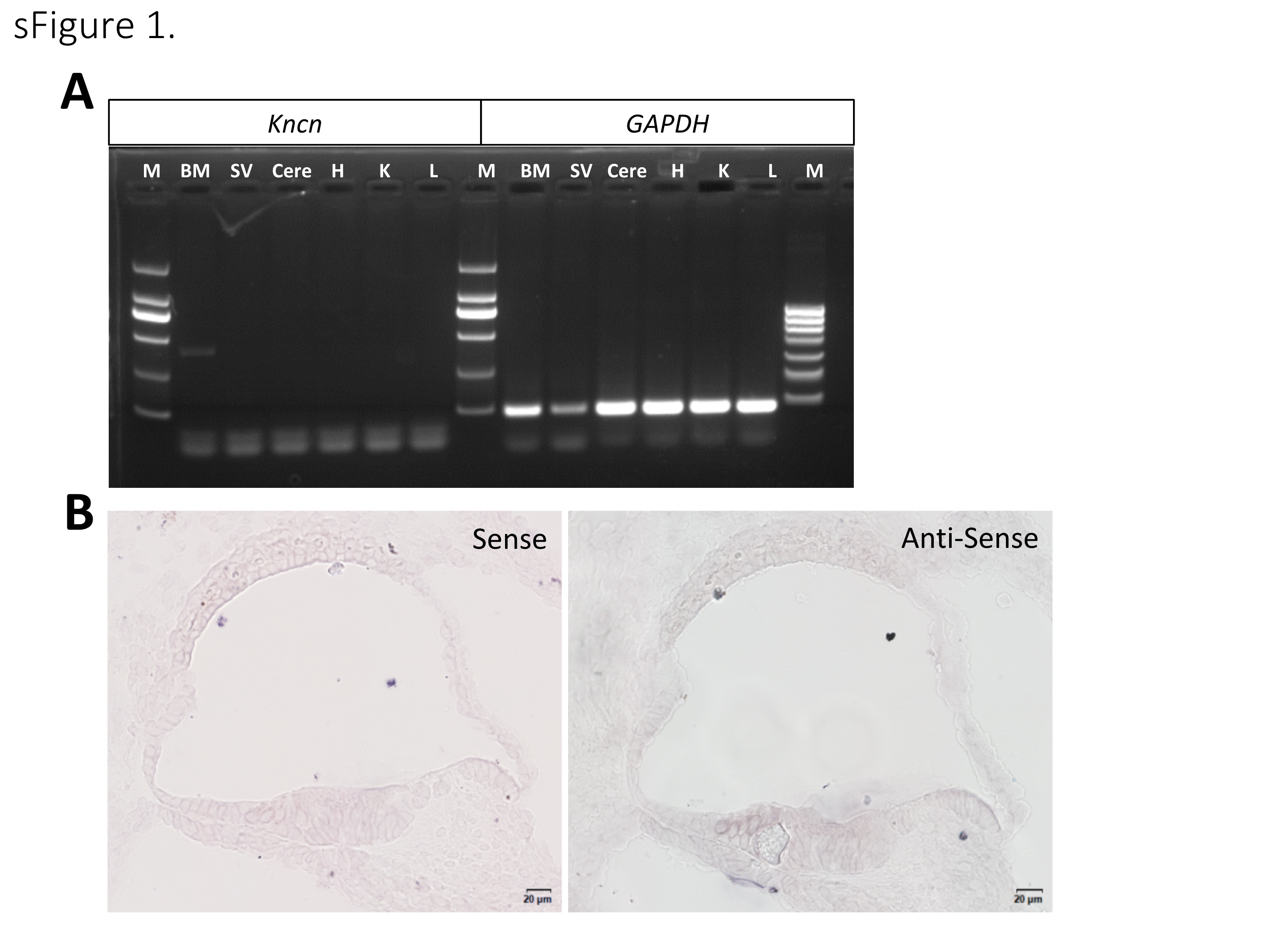

Supplement: FIGURE S1 — Analysis of Kncn expression. (A) RT-PCR study of Kncn mRNA level in different tissues from P7 mice. The positive band for Kncn was 405bp. Abbreviation: M, maker; BM, basilar membrane; SV, stria vascularis; Cere, cerebellum; H, heart; K, kidney; L, liver. (B) ISH analysis of Kncn mRNA expression in cochlear sections from P4 mice. Panels showed the sense and anti-sense probe staining in the organ of Corti and hair cells. [file Image_1.TIFF]
