# Supplementary material for: Deletion of Kncn Does Not Affect Kinocilium and Stereocilia Bundle Morphogenesis and Mechanotransduction in Cochlear Hair Cells
Source: Front Mol Neurosci. 2018 Sep 11;11:326. doi: 10.3389/fnmol.2018.00326 (PMC6141681; doi:10.3389/fnmol.2018.00326)
Supplement: TABLE S1 — List of primers used in the study. [file Table_1.DOCX]

Supplemental Table 1

| Kncn-sgRNA-F | TTAATACGACTCACTATAGGgctgccggccaccaggccgaGTTTTAGAGCTAGAAATAGC |
| --- | --- |
| Kncn-sgRNA-R | AAAAGCACCGACTCGGTGCC |
| Genotyping forward primer | 5’-GACCCACGTCACTAGAACCC-3’ |
| Genotyping reverse primer | 5’-CCAGCACACTCACTCAACCA-3’ |
| RT-PCR forward primer (for sFig. 1A) | 5’-ATGGACATCCCCATCAGCACCA-3’ |
| RT-PCR reverse primer (for sFig. 1A) | 5’-TTAGCCCTCCTCAGTTCCCCG-3’ |
| Q-PCR forward primer (for Fig.1A,B) | 5’-GGCTCCTCATCTTCGCCTAC-3’ |
| Q-PCR reverse primer (for Fig. 1A,B) | 5’-CCATGGTCTGGTCCTGAGTT-3’ |
| Kncn-ISH-F | 5’-TAATACGACTCACTATAGGGAAATGTGGCCAGGCAGGAAC-3’ |
| Kncn-ISH-R | 5’-CATTAACCCTCACTAAAGGGAACTCAGGTTTCCTATGGCAGGT-3’ |
